# Prognostic Value of Motor Timing in Treatment Outcome in Patients With Alcohol- and/or Cocaine Use Disorder in a Rehabilitation Program

**DOI:** 10.3389/fpsyg.2018.01945

**Published:** 2018-10-22

**Authors:** Susanne Yvette Young, Martin Kidd, Jacques J. M. van Hoof, Soraya Seedat

**Affiliations:** ^1^Department of Psychiatry, Faculty of Medicine and Health Sciences, Stellenbosch University, Stellenbosch, South Africa; ^2^Centre for Statistical Consultation, Statistics and Actuarial Sciences, Stellenbosch University, Stellenbosch, South Africa; ^3^Department of Psychiatry, Radboud University Medical Centre, Nijmegen, Netherlands

**Keywords:** motor timing, prognostic value, temporal cognition, movement, substance use disorder, cocaine, alcoholism

## Abstract

**Introduction:** Individuals with Substance Use Disorder (SUD) often have cognitive deficits in multiple domains, including motor timing deficits, with recovery times of up to 1 year. Cognitive deficits influence treatment outcomes and abstinence. To our knowledge, timing deficits have not been investigated with regard to treatment outcome and relapse.

**Methods:** This prospective study tested the prognostic value of motor timing in SUD with regard to treatment outcome. The study sample consisted of 74 abstinent in-patients at a private treatment programme for drug/alcohol dependence at the Momentum Mental Healthcare clinic in Somerset West, South Africa, diagnosed with alcohol and/or cocaine dependence. Participants were tested at three points: (i) Within 72 hours of the start of the treatment programme (ii) after completion of the treatment programme at 8 weeks (measure of treatment response) through filling out self-report questionnaires and experimental motor task testing, and (iii) a third visit followed through a telephonic interview at 12-months (measure of relapse).

**Results:** Motor timing alone predicted 27 percent of the variance in alcohol self-efficacy score change, and 25 percent variance in cocaine self-efficacy change scores at treatment completion. Specifically, spatial errors, synchronization errors and inter- response interval errors of a spatial tapping task at baseline predicted self-efficacy in alcohol self-efficacy. Cocaine self-efficacy was predicted by spatial errors and contact times of a spatial tapping task at very high tempi (300 ms) only. The high rate of dropout at 12 months post-treatment did not allow for further analysis of the prognostic value of motor timing on relapse.

**Conclusions:** The results of this investigation show us that motor timing holds prognostic value with regard to treatment outcomes. Motor timing predictors for relapse require further investigation going forward.

## Introduction

Alcohol and cocaine are amongst the most widely abused substances (The Global Drug Survey 2015 Findings, 2015). Chronic exposure to substances leads to structural and functional brain disturbances (Moselhy et al., 2001; Oscar-Berman and Marinkovic, 2003; Scheurich, 2005; Verdejo-García et al., 2007; Volkow et al., 2010; Bühler and Mann, 2011), which underlie the cognitive decline and behavioral changes found in Substance Use Disorder (SUD) (Miller, 1991; Bates et al., 2002; Goldstein and Volkow, 2011). Recent studies on the neurocognitive effects of long-term substances of abuse show that, instead of specific impairments, dysfunctions occur for a wide array of cognitive domains (Spronk et al., 2013; Stavro et al., 2013). One such domain is motor timing abilities (Wittmann et al., 2007). Motor timing is defined as the ability to organize movement according to temporal structures. One of the few studies to date that attempted to examine motor timing in stimulant dependent individuals, whilst controlling for possible confounds, found that motor timing deficits are present in this population (Wittmann et al., 2007). The stimulant dependent group showed abnormal motor timing abilities on all timing tasks, except sensorimotor synchronization.

The direct influence of these functional deficits on recovery and sobriety of individuals with SUD remains unclear (Bates et al., 2002). Long-lasting changes in brain regions are shown to contribute to relapse, which can occur weeks, months, and even years after substance use (Welberg, 2011). There are few methods to measure the success of SUD treatment outcomes. Self-efficacy, is considered an important indicator in the management of SUDs and in treatment outcome more specifically (Maisto et al., 2000; Burleson and Kaminer, 2005; Ilgen et al., 2005; Dolan et al., 2008; Kadden and Litt, 2011), and defined as an individual's confidence in his/her ability to abstain from certain adverse behaviors, such as substance use (Bandura, 1994). Self-efficacy is seen as an important factor in predicting behavior related to health, the successful application of coping mechanisms (Tate et al., 2008), and changing unwanted behavior (Sheeran et al., 2016). Studies have shown that increased self-efficacy is related to the ability to suppress habitual responses, a higher level of well-being, the ability to achieve complete abstinence after treatment, to apply healthier coping mechanisms, increase participation in aftercare, predict the duration of abstinence, and decrease the use of alcohol and other substance use after treatment (Vielva and Iraurgi, 2001; McKay et al., 2003; Warren et al., 2007; Tate et al., 2008). Increased levels of self-efficacy at treatment admission, discharge, and 1 month after treatment was found to be a strong predictor of prolonged abstinence (Coon et al., 1998; Ilgen et al., 2005; Dolan et al., 2008; Kadden and Litt, 2011).

Amongst the more objective measures are blood or urine tests. However, not every treatment setting allows for such measures to be used in a useful way, requiring compromises to achieve the most valid outcome possible. In an inpatient treatment programme, criteria such as abstinence and retention are fulfilled by most, if not all inpatients, and are not necessarily an indication of treatment success or a guarantee of abstinence. In this case a more subjective measure, such as self-reported belief in the ability to abstain is an acceptable measure.

In sum, individuals with SUD often have cognitive deficits in multiple domains, with recovery times of up to 1 year (Spronk et al., 2013; Stavro et al., 2013). These deficits influence treatment outcomes and abstinence (Pitel et al., 2007; Fox et al., 2008). In addition, motor timing deficits have been found in SUD (Wittmann et al., 2007) but, to our knowledge, timing deficits have not been investigated with regard to treatment outcomes. Early detection of motor timing deficits may be predictive of treatment outcomes. Owing to the limited number of pharmacological treatment options, many clinicians worldwide rely solely on psychosocial approaches (Dackis and O'Brien, 2001). Cognitive deficits experienced by individuals with SUD may, therefore, be of broad relevance in psychosocial adaptation, and more specialized research that informs clinical practice and guides future research is needed to improve and broaden treatment options. This prospective study tested the theoretical basis for prognostic indicators in SUD with regard to motor timing (measured in terms of treatment response and relapse). We expected that (i) the capacity to structure, organize and plan an action directly toward a visual target [motor reaction task (Task 1)]; (ii) cognitive control [Go-nogo task (Task 3)]; and (iii) synchronization abilities [Spatial-tapping task (Task 2)] would be prognostic of treatment outcome (self-perceived self-efficacy to abstain from substances) at 8 weeks and possible relapse (dichotomised as “yes/no”).

## Methods

### Sample

The study sample consisted of 74 abstinent patients, aged 18–60 years, and diagnosed with alcohol and/or cocaine dependence. Patients with a primary diagnosis of alcohol and/or cocaine dependence who were detoxified were included. Patients who met criteria for other substance abuse (lifetime or current) were included, provided that these were not their *primary* drugs of use/abuse. Patients who met criteria for other substance dependence (i.e., other than cocaine/alcohol) were excluded. For the alcohol group, patients were excluded if they had a current or past history of dependence on cocaine. For the cocaine group, patients with a current or past history of alcohol dependence were excluded.

### Procedures

Participants were all inpatients at a private treatment programme for drug/alcohol dependence at a treatment clinic in Somerset West, South Africa. The clinic offers treatment to individuals who are mainly of Dutch nationality as the main patient referral company is situated in the Netherlands. The comprehensive primary care treatment program, which formed the standard of care for all participants, centers on an 8-week cycle of treatment comprising group therapies, individual counseling, written work and a psycho-educational lecture series. All participants worked individually with a therapist. A full medical examination was conducted on every patient included. This consisted of a physical examination and toxicology and biochemistry work-up by the psychiatric nursing staff.

Participants were tested at three points in time: (i) within 72 hrs of the start of the treatment programme, (ii) after completion of the treatment programme at 8 weeks (measure of treatment response), and (iii) at the 12-month follow-up period (measure of relapse). Designated counselors at the clinic enquired from patients about their potential interest in participating in the study. Only participants who gave written consent and who were eligible upon screening were invited for a first research visit. After written consent was obtained, participants were enrolled for participation. Two study visits were conducted at the clinic. Each of these visits entailed filling out self-report questionnaires and experimental motor task testing. During baseline assessments a socio-demographic questionnaire, the *Measurements in the Addictions for Triage and Evaluation.2* (MATE.2.10) (Schippers et al., 2010), the *Mini International Neuropsychiatric Interview version 5* (MINI 5) (Lecrubier et al., 1997), the *Edinburgh Handedness Questionnaire* (EHQ) (Büsch et al., 2010), The *Alcohol Use Disorders Identification Test* (AUDIT) (Lundin et al., 2015), and *Drug Use Disorders Identification Test* (DUDIT), (Hildebrand, 2015), the *Sheehan Disability Scale* (SDS)(Beck et al., 2004), and the *Beck Depression Inventory* (BDI) (Beck et al., 1988), and the *Alcohol Abstinence Self-Efficacy Scale* (AASE) and the *Cocaine Abstinence Self-Efficacy Scale* (CASE) (DiClemente et al., 1994), the *Short Alcohol Withdrawal Scale* (SAWS) (Gossop et al., 2002), and a motor task battery (see section Temporal Processing: Action-Based Timing Tasks) were administered. During the second visit (at treatment completion) the MATE.2.10, SDS, BDI, AASE, CASE were repeated. All assessments were conducted in a structured manner by either the principal investigator or a trained research assistant. One research assistant was appointed for a period of 2 years. For quality control, all questionnaires and task performance scores, including data entry, were cross checked by both the PI and the research assistant. For the administration of all assessments, standard operating procedures were followed. Task instructions were read out in the same way to each participant. The same order of assessment was used for each visit and for each participant. After completion of the first visit, an appointment for a second assessment was made. Assessments were undertaken within 72 hrs of initiation (visit 1) of the treatment program and repeated at the end of the 8 weeks (last 72 hrs, visit 2). A telephonic interview using the MATE.2.10 (Schippers et al., 2010) was administered at 12 months to assess relapse. The research team did not stay in contact with the patient during the time between discharge and follow up, due to patient privacy policies of the clinic. All data were de-identified and kept confidential. In order to encourage honesty patients were reminded that none of test results were to be shared with clinical staff.

### Measures

Gender, age, handedness, ethnicity, education, family history of substance dependence, previous admissions/counseling/therapy history, symptoms of disability, and drug or alcohol usage (including last intoxication, last drink and last withdrawal), depression, and psychopathology were assessed with a self-administered demographic questionnaire, the EHQ (Büsch et al., 2010), The MATE.2.10 (Schippers et al., 2010), MINI 5 (Lecrubier et al., 1997), AUDIT (Lundin et al., 2015), and DUDIT (Hildebrand, 2015), the SDS (Beck et al., 2004) the BDI (Beck et al., 1988) and the SAWS (Gossop et al., 2002).

#### Self-efficacy

The AASE and CASE (DiClemente et al., 1994) are both self-report questionnaires consisting of 20 questions that give an indication of the degree of self-efficacy to abstain from substance use (i.e., the confidence to abstain from alcohol and / or cocaine). Items have a 5 point Likert scale ranging from not at all (1) to very much (5) for example, the level of temptation that a person experiences to use a substance in a specific situation like when he/she is concerned about someone. Four subscales can be distinguished (1) social situations, (2) negative affect, (3) positive emotions, and (4) physical or other worries (DiClemente et al., 1994). For a total score, all items are added up and divided by the number of questions (20).

#### Temporal processing: action-based timing tasks

The motor tasks consisted of a series of reaction-prediction visuo-motor pointing tasks to measure different aspects of motor timing (motor sequencing, synchronization, and decision-making). The sequential pointing tasks were all designed by Professor Y. Delevoye-Turrell and her team at the University of Lille, France. These tasks have been used in previous research but not SUD research, nor in prognostic research of any kind previously (Delevoye-Turrell et al., 2007, 2012; Dione et al., 2013; Dione, 2014; Dione and Delevoye-Turrell, 2015). For testing, participants were seated in a chair in front of a tactile screen (Elo Touch) of 53 cm by 36 cm by 30 cm. The flat resting screen was placed horizontally and in close proximity to the participants' midline in order to avoid muscle fatigue from the repetitive pointing movements. Visual and auditory signals were controlled via a PC with coded software in C++. For a detailed overview of these tasks, please see protocol publication (Young et al., 2016).

##### Reactivity: the motor reaction task

Motor sequencing abilities were evaluated using a simple finger-pointing task to visual dots presented on the touch screen. Participants are required to lift (action initiation- measured as Reaction Time), and touch (action execution- measured as Movement Time), one dot (condition one,) a series of two (condition 2), or three dots (condition 3).

The manipulation of the complexity (the number of dots) of the motor sequence provided the means to assess lower order timing mechanisms (one target) and higher order mechanisms (2 and 3 dots) through the capacity of participants to structure, organize, and plan an action through time and space by ensuring accurate pointing in combination with fast movements. Condition 1 is designed to measure lower order mechanisms of movement initiation and execution, whereas condition 2 and 3 are designed to measure higher order mechanisms through increased complexity requiring structuring and planning of motor timing. Participants are instructed to start with the index finger of the dominant hand placed on the square starting zone which is situated at the bottom left edge of the screen. As soon as a black dot appears on the screen, the task is to lift off from the target (square) and touch the target(s) as fast as possible. Three levels of complexity are counterbalanced: one target, two-target or three-target conditions.

##### Synchronization: the spatial-tapping task

**Figure 1:**
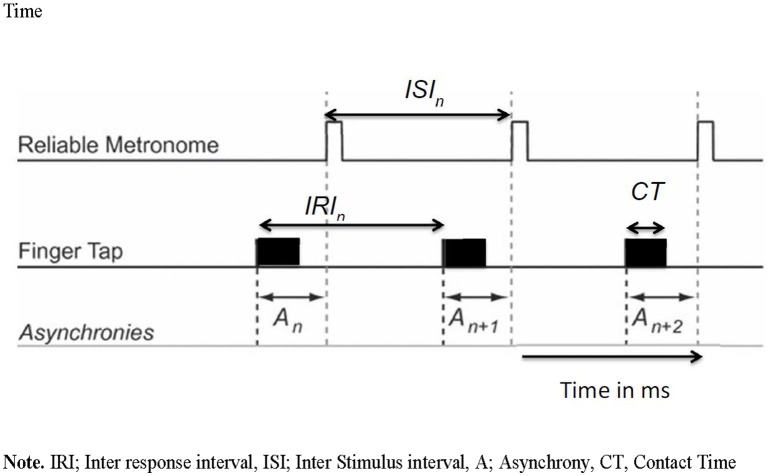
Viaual overview of inter response interval, inter stimulus interval, asynchrony and contact time. IRI, Inter response interval; ISI, Inter stimulus interval; A, Asynchrony; CT, Contact Time.

With this task, we aimed to evaluate how well self-initiated actions to external stimuli, present in the environment, are timed (synchronized) using a Spatial-tapping task (Dione, 2014). This task measures pointing accuracy in time and space as well as error in fluency and accuracy. On the tactile screen display are six black dots 100 mm apart in a circle. The task is to touch each target, one after the other, starting from the bottom right target, and moving counter-clockwise using the right index finger (fist closed). The tempo of the external rhythm is fixed in terms of inter stimulus interval (ISI) and is considered an important independent variable in timing research. Each condition is constituted of a series of sixty taps of, in total, 5 trials (ISI = 1100 ms; 700, 500, 400, and 300 ms). The total duration of the task is approximately 10 min. In each trial, participants are presented with an auditory rhythm that must be used to pace their actions. After listening to the tones for 5.5 s, participants start tapping for a total trial duration of 35 s. Timing performances on this task were measured through inter-response interval errors (IRI error) and synchronization errors (Asynchrony). The IRI was measured as the time intervals between the start of two successive taps. The IRI error was then computed as the percentage of absolute difference between each IRI and the reference ISI of a given trial. Asynchrony was measured through the difference between onset of a tap and the time of onset in the external rhythm. Spatial performances were measured through the measurement of endpoint distributions of pointing actions and were plotted as a function of each visual target position. The mean spatial error (SE) of these spatial ellipses were used as an indication of spatial performances. The control of pauses was measured through contact time (CT) and defined as the time of finger contact with the touch screen. This measure (in ms) was used as an indicator of the amount of voluntary pauses in the gesture. See Figure 1 for an overview of how IRI errors, CT, and Asynchrony were measured.

##### Cognitive control: the go-nogo task

A modified version of the Go-nogo paradigm was designed to measure reaction times through a tactile touch of the touch screen. The starting zone is situated at the bottom left edge of the screen. The target is a white circle with a black letter or one-digit black number and participants are instructed to act as fast as possible (Go) or to refrain from acting (Nogo), depending in the condition of the task. In the first condition, the task is to tap the target that appears as fast as possible (100% Go). In the following blocks, participants are instructed to react and tap the target as fast as possible, but only if the target is a letter (50% Go). If the target is a number, they are to refrain from reacting (Nogo). Numbers and letters were presented in semi-random order. The targets were presented for 5 s on the screen, with a random phase lag of ±300 ms in order to avoid anticipatory responses. Cognitive control was measured through decision making (by measuring reaction times based on the participant's response directly *after* a Go target or after a Nogo target) and adaptability (by measuring reaction times on responses on targets that came directly *after* a Nogo Target Error).

### Data analyses

Backward step-wise regressions were conducted to establish the best fit of motor timing variables regarding their predictive power on self-efficacy total score change at 8 weeks. Best subset regressions were used to select the best fitting models out of the top 20 models with the least number of predictor variables.

## Results

### Sample

#### Demographics

All participants included in this study completed treatment. All participants were right handed, (*n* = 74), 80 percent were male, and the mean age was 36.6 years old (*SD* = 10.5, *mode* = 27, range 19–60). Forty-two participants (59%) were employed, and 27 participants (36.5%) were receiving unemployment benefits. Half of the participants were single, 13 participants (20%) were divorced and 28 participants (40%) had children.

#### Clinical characteristics

Patients with comorbid disorders, as assessed on the MINI 5, at the beginning of their treatment were excluded from entry into the study; however, at discharge (8 weeks), some participants had been diagnosed by their treating clinicians, during the course of treatment, with comorbid disorders (*n* = 10, 15% Axis 1 Psychiatric disorders; *n* = 15, 20% Axis II Personality Disorders; *n* = 5, 7% both Axis 1 and 2). Previous outpatient treatment had been attempted unsuccessfully by 38 participants (51.4%) while 23 participants (31%) had received psychotherapy, 12 participants (16.2%) had previously been admitted to psychiatric inpatient care (non SUD- majority due to a failed suicide attempt), and for 21 participants (29%), this was the second (or more) attempted inpatient rehabilitation. All patients were detoxified before treatment. However, upon admission, 23 (31%) of the participants had a positive alcohol test (through a breathalyzer examination) while 38 participants (54%) had a positive drug test (cocaine *n* = 25 (33%), benzodiazepine *n* = 8 (10.8%), cannabis *n* = 5 (6.8%), and amphetamine *n* = 1 (1.4%). Craving symptoms were minimal at baseline (MATE Q1 cut off scores of <12 are considered minimal craving) (*m* = 7.5, *SD* = 3.9). Withdrawal symptoms at admission were minimal on average (*m* = 8.66, *SD* = 6.5, *Mdn* = 7), however a minority of participants suffered from moderate to severe withdrawal (cut off score for minimal withdrawal <12, *CI* = 0;30). Drug use other than cocaine and/or alcohol was minimal, with 9 percent using ecstasy, other stimulants (e.g., Speed, Methamphetamine, 15 percent) and sedatives (12 percent) in the 30 days before admission. The severity of psychiatric comorbid symptoms was below threshold on the Anxiety, Depression and Stress scale (MATE Q2 total score of <60) (*m* = 41.8, *SD* = 25.2, mode = 12). A detailed overview of the clinical and demographic results can be found in Table 1.

**Table 1:** Demographic and clinical characteristics of the separate groups and all patients combined.

***N* = 74**	**Alcohol**		**Cocaine**		**Alcohol/cocaine**		**All patients**	
	***n** = **25***		***n** = **24***		***n** = **25***		***n** = **74***	
	***M***	***SD***	***M***	***SD***	***M***	***SD***	***M***	***SD***
Age	44.2	0.8	32.8	0.9	32.8	0.9	37.1	11.1
Alcohol use last 30 days	18.5	10.1	4	5.3	16.6	8.3	13.1	10.3
Alcohol quantity used last 30 days (units)	15.2	9.6	5.7	7.4	16.4	15.6	12.6	12.3
Cocaine use last 30 days	10.8	0.2	15.3	10.6	10.8	9.7	8.8	10.4
Cocaine quantity used last 30 days (grams)	0.04	0.1	3.2	3.4	1.3	1.4	1.5	2.5
AUDIT	25.3	8.9	8.2	5	24.6	7.5	19.6	10.7
DUDIT	7	8.1	31.5	7.6	28.6	8.8	23.6	13.2
Sheehan Disability Scale	16	7.1	21.4	6.3	17.4	9	18.6	7.8
Duration of Use	24.2	11.9	12.4	7.6	15.2	8.3	17.3	10.3
Age of first Use	20.5	9.3	20.9	5.5	17.3	4.1	19.6	6.8
Abstinence in days	16	14.1	14.6	12.1	14.2	10.43	14.9	12.2
GAF score at admission	52.8	6.9	51.5	11.7	52.5	7.5	52.3	8.9
Physical complaints	12.2	6.8	9.1	8.4	11.6	7.7	11	7.6
Craving (last 30 days)	6.7	3.3	7.9	4.2	7.8	4.2	7.5	3.9
Comorbid symptom severity	19.1	12.9	21	11.7	22.3	13.5	20.9	12.6

*Results of separate groups; Intelligence: Nederlandse Leestest voor Volwassenen, AUDIT, Alcohol Use Disorder Identification Test; DUDIT, Drug Use Disorder Identification Test; Quality of Life, Sheehan Disability Scale (SDS), GAF, Global Assessment of Functioning. Physical complaints MATE 5; Measurements in the Addictions for Triage and Evaluation, Physical Complaints/health related symptoms (withdrawal) in the last 30 days; Craving MATE Q1 Measurements in the Addictions for Triage and Evaluation, Craving Scale regarding the last 30 days; Comorbid symptom severity MATE Q2, Measurements in the Addictions for Triage and Evaluation, Anxiety, Depression, Stress Scale last 30 days*.

### Main results: treatment outcomes

#### Self-efficacy to abstain from alcohol use

An overview of the timing task results can be found in Table 2. A best subset regression analysis of all motor tasks showed that motor timing deficits at baseline hold prognostic value with regard to self-efficacy to abstain from alcohol use (*R*^2^ = 0.27). Both the Motor Reaction task and the Go-nogo timing task were not predictive of self-reported self-efficacy to abstain from alcohol use. Of the Spatial Tapping Task, SE (at ISI 300 ms) at baseline were predictive of total change in percentages in self-reported self-efficacy to abstain from alcohol use (*b* = −0.26, *t*_(50)_ = −2.05, *p* = 0.04). Furthermore, Asynchrony of the Spatial Tapping Task was found predictive of change in alcohol self-efficacy scores at discharge. Asynchrony (at ISI 400 ms) of the Spatial Tapping Task at baseline were predictive of total change in alcohol self-efficacy scores (*b* = −0.37, *t*_(50)_ = −2.14, *p* = 0.03). IRI of the Spatial Tapping Task were also found to be predictive of alcohol self-efficacy to abstain from alcohol use at ISI 500 ms intervals (*b* = −0.28, *t*_(50)_ = −2.10, *p* = 0.04) and ISI 700 ms intervals (*b* = −0.28, *t*_(50)_ = −2.01, *p* = 0.04). Although not statistically significant, Asynchrony and IRI errors of the Spatial Tapping task at the 1100 ms interval conditions occurred in 20 and 17 times, respectively, in the top 20 best predictor models.

**Table 2:** Means and standard deviations of all motor tasks performances of patient group comparisons at baseline.

***N* = 74**			**Alcohol**		**Cocaine**		**Alcohol/cocaine**		**All patients**	
			***n** = **25***		***n** = **24***		***n** = **25***		***n** = **74***	
			***M***	***SD***	***M***	***SD***	***M***	***SD***	***M***	***SD***
Go-nogo task	Reaction time	After go	0.66	0.51	0.42	0.04	0.51	0.18	0.51	0.01
		After nogo	1.28	1.74	0.65	0.41	0.68	0.45	0.59	0.11
		After nogo error	0.47	0.72	0.24	0.27	0.35	0.34	0.28	0.03
Finger Tapping Task		Spontaneous ISI	494.85	140.58	401.05	99.26	451.53	120.90	451.53	120.90
	Space	Contact time (ms)	0.15	0.07	0.11	0.02	0.15	0.04	0.14	0.01
		Spatial error (%)	11.3	3.3	13.7	3.7	11.7	2.7	12.2	0.41
	Interval timing Time	Asynchrony (%)	−0.05	0.08	−0.03	0.06	−0.04	0.07	−0.04	0.01
		IRI error (%)	6.7	2.3	7.1	3.2	6.8	2.8	7.1	0.18
	Space	Contact time (ms)	0.20	0.13	0.16	0.10	0.19	0.12	0.18	0.01
		Spatial error (%)	11.4	3.5	11.6	3.2	11.1	3.2	11.6	0.22
Motor reaction task	Movement Initiation	1 target (ms)	0.40	0.07	0.41	0.08	0.40	0.06	0.40	0.01
		2 targets (ms)	0.41	0.06	0.41	0.07	0.41	0.06	0.41	0.01
		3 targets (ms)	0.40	0.05	0.41	0.07	0.40	0.03	0.40	0.01
		All targets (ms)	0.40	0.06	0.41	0.06	0.40	0.07	0.40	0.01
	Execution	1 target(ms)	0.36	0.09	0.38	0.09	0.43	0.13	0.39	0.01
		2 targets(ms)	0.34	0.07	0.33	0.08	0.35	0.07	0.33	0.01
		3 targets(ms)	0.34	0.07	0.31	0.07	0.33	0.07	0.32	0.01
		All targets (ms)	0.35	0.08	0.36	0.10	0.33	0.08	0.35	0.01

#### Self-efficacy to abstain from cocaine use

A best subset regression analysis showed that motor timing deficits at baseline hold prognostic value with regard to self-efficacy to abstain from cocaine use (*R*^2^ = 0.25). Both the Motor Reaction task and the Go-nogo timing task were not predictive of self-reported self-efficacy to abstain from cocaine use. SE of the Spatial Tapping Task at 300 ms intervals (*b* = −0.31, *t*_(50)_ = 2.62, *p* = 0.01) and at 500 ms intervals (*b* = 0.36, *t*_(50)_ = 2.69, *p* < 0.01) at baseline were predictive of total change in percentages in self-reported self-efficacy to abstain from cocaine. CT of the Spatial Tapping Task at 300 ms intervals were also found to be predictive of total change in cocaine self-efficacy (*b* = 0.31, *t*_(50)_ = −2.62, *p* = 0.01). Although not significant, Asynchrony of the Spatial Tapping Task at 300 ms interval condition occurred in 17 of the top 20 best predictor models.

#### Prognostic value of motor timing in relapse prediction

Of the 74 participants, 44 were interviewed at 12-months post-discharge, with 30 participants lost to follow up. Data from 36 participants with the least missing data were used for these analyses. Of these 36, 6 relapsed while all other participants remained abstinent of drugs and alcohol use post-discharge. The small sample, and limited power, precluded analysis of motor timing predictors of relapse.

## Discussion

The main aim was to test for prognostic indicators in SUD with regard to motor timing (measured in terms of treatment response). We expected that motor coordination and planning abilities, synchronization abilities and decision making would be prognostic of treatment outcomes (self-perceived efficacy to abstain from substances) at 8 weeks and relapse at 12 months (yes/no). With regard to treatment outcomes, we found that only the Spatial Tapping Task variables were predictive, and explained 27% of alcohol use self-efficacy, and 25% of cocaine use self-efficacy at discharge.

With regard to alcohol self-efficacy, SE (at ISI 300 ms), Asynchrony (at ISI 400 ms) and IRI Errors (at ISI 500 and 700 ms) were predictive of self-perceived self-control to abstain from alcohol use. With regard to cocaine self-efficacy, SE (at ISI 300 and 500ms) and CT (at ISI 300) were predictive of self-perceived self-control to abstain from cocaine use. Due to the very small number of participants who could be reached for follow-up, the analyses of motor timing variables with regard to relapse at 12 months were omitted.

Interestingly the motor timing variables predicting cocaine and alcohol self-efficacy were not the same. This may indicate that there are different factors at play in different SUDs. The only timing variable that was shared by both alcohol and cocaine self-efficacy, and both at high tempi only, was SE on the Spatial Tapping Task. Spatial abilities rely heavily on visual feedback and patients may choose to be accurate above being correct which could point to high compulsivity levels in patients. What the predicting variables have in common is that they are all at high tempi. This, again, may point to deficits that only manifest when patients are under pressure, namely when the cognitive load goes up, which is the case when time constraints are present, deficits become apparent.

Another interesting assumption that can be made, based on our findings, is the overlap between millisecond timing and SUD deficits found in brain circuitry. The literature suggests that the use of substances is associated with deficits in frontal lobe and striatal functioning (Moselhy et al., 2001; Spronk et al., 2013) through alteration in activation of the cortico-limbic reward circuit (Welberg, 2011). Aspects of self-control, delayed self-gratification, drive inhibition and anticipation of the consequences all require the functional integrity of executive pre-frontal cortical system (Lyvers, 2000). The breakdown of orbitofrontal cortical communication may, in part, explain the decrease in motivation and self-control experienced in individuals with SUD (Dackis and O'Brien, 2001; Welberg, 2011). A recent study examining brain circuits involved in time perception in the millisecond and second ranges probed the role of the right supplementary motor area (SMA), the right dorsolateral prefrontal cortex (dlPFC), and the cerebellum (Méndez et al., 2017). Researchers temporarily altered activity in healthy participants using transcranial magnetic stimulation with the continuous Theta Burst Stimulation (cTBS) protocol. Participants were tested on a temporal categorization task before and after stimulation using intervals in the hundreds and thousands of milliseconds ranges, as well as on a pitch categorization task, used as a further control. Researchers looked for changes in the Constant Error and the Relative Threshold, which, respectively, reflect participants' accuracy at setting an interval that acts as a boundary between categories and their sensitivity to interval duration. The researchers found that after cTBS in all of the studied regions, the Relative Threshold, but not the Constant Error, was affected, and only when hundreds of milliseconds intervals were being categorized. Categorization of pitch, and thousands of milliseconds intervals were not affected. These results suggest that the frontocerebellar circuit is particularly involved in the estimation of intervals in the hundreds of milliseconds range (Méndez et al., 2017). This overlap in brain circuitry is affected by SUD, and motor timing in the millisecond range may indeed hold promise for future research focusing on biomarkers of SUD or indicators of the severity of damage due to substance abuse.

One explanation of how motor timing deficits could contribute directly to higher predisposition for relapse in addiction is proposed by van Hoof (2002, 2003). The model explains that the motoric mechanisms necessary for grasping stationary and moving objects evolved and matured to organize cognitive and emotional processes, such as affiliation and intimidation. This organizational process resulted in the capacity to organize intentional behavior van Hoof (2002, 2003). Thus, mental representations of intended or goal-action effects are responsible for the planning and execution of appropriate movements required to achieve a goal van Hoof (2002, 2003). Following this model, major psychiatric disorders (e.g., schizophrenia and SUDs) may be understood as manifestations of imbalances between an automatic mode of action (referred to as the Drive Mechanism) and a more cognitive-predictive mode of action (referred to as the Guidance Mechanism, GM). This bimodal distribution and evolutionary neurobiological model may provide a useful pathogenic framework for the classification of major psychiatric disorders, including SUDs van Hoof (2002, 2003), and is tested as part of ongoing investigation (Young et al., 2016).

Several limitations warrant mention. First, the high attrition rate at the 12-month follow-up precluded the analysis of predictors of relapse. The high rate of attrition may have been mitigated by a shorter time to follow-up and the use of face-to-face structured interviews rather than telephonic interviews, supplemented by urine drug testing, to confirm abstinence. Another limitation of the study was the use of a subjective (self-reported self-efficacy) rather than more objective measures available. As mentioned previously, among the more objective measures are blood or urine tests. However not every treatment setting allows for such measures to be used in a useful way, requiring compromises to achieve the most valid outcome possible. The validity of treatment outcome measures in research depend on the type of treatment that patients are undergoing. Even though lacking in objectivity, self-efficacy is a subjective but an acceptable measure of treatment outcome in our research setting. The study of treatment success in an inpatient, closed-off, treatment setting precludes the assessment of more objective outcomes, such as retention and abstinence. Retention and abstinence are achieved by most in these settings, which, if used as indicators would give the false impression of greater treatment success. However, due to the subjective nature of the outcome measures used the results should be interpreted with care. Another limitation is that participants without comorbid disorders and partcipants who did not use psychotropic medications, at baseline, were included in the study, in order to avoid the confounding effects of comorbid psychopathology and the effects of psychotropic medications on motor timing performance. While this may reduce the generalizability of these findings to patients with SUD and comorbid psychopathology, even though we excluded patients with a comorbid disorder at baseline, by the end of treatment more than a third of the sample had been diagnosed with comorbid disorders by their treating clinicians. This is not unexpected given that (i) dual diagnosis is highly prevalent in this population and (ii) when patients with SUD enter treatment, it is often necessary to observe them after an extended period of abstinence in order to distinguish between the effects of substance withdrawal (which can be prolonged) and the symptoms of comorbid mental disorders. In examining baseline predictors of relapse, comorbid disorders were not adjusted for in the analyses. This poses another question: is it the comorbid disorder that may have had mediating effects? Another limitation is that patients were in treatment for a period of 8 weeks. During this period, they did not have access to their phones, ate healthily, exercised, engaged in a structured programme in a supported and therapeutic milieu, and did not face usual life stressors. This “stability” of environment may have impacted on the findings of our research. Research attempting to replicate the results in outpatient populations may shed light on this possible limitation.

Another limitation was that even though patients were detoxified before arrival at the clinic some of them still tested positive for substances. The clinic which Dutch patients were admitted to is situated in South Africa; however, the long trip to SA may have resulted in some patients using substances during their travels. This means that a number of patients may have undergone another withdrawal during their stay in the clinic. Even though withdrawal and craving were well below cut-off scores, some still experienced moderate to severe symptoms, such as tremor which may have influenced performance on the motor tasks. This limitation may have influenced the results of this study.

Future research should focus on more diverse populations with SUD and on inpatients and outpatients who are at different points in their recovery process. A possible explanation for the association between cognitive load and motor timing abilities in SUD patients suggests that time constraints and errors may be perceived as (more) stressful; they also increase (perceived) cognitive load and subsequently lead to loss of control over inhibition and rhythmic abilities. To our knowledge, this is the first study to demonstrate such an association, and based on our findings, replication studies on motor timing abilities in SUD samples, their prognostic value and their specificity for different SUD, are warranted.

## Availability of data and materials

The raw data and materials supporting the conclusions of this manuscript will be made available by the authors, without undue reservation, to any qualified researcher.

## Ethics statement

This study was carried out in accordance with the recommendations of Declaration of Helsinki and the South African Guidelines for Good Clinical Practice, University of Stellenbosch's Health Research Ethics Committee. The protocol was approved by the University of Stellenbosch's Health Research Ethics Committee. All subjects gave written informed consent in accordance with the Declaration of Helsinki.

## Author contributions

SY: Has made substantial contributions to the conception and design, acquisition of data, and analysis and interpretation of data. SY has been involved in drafting the manuscript and revising it critically for important intellectual content. SS: Has made substantial contributions to the conception and design, analysis and interpretation of data. SS has been involved in drafting the manuscript and revising it critically for important intellectual content. SS provided final approval of the version to be published. MK: Has made substantial contributions to the statistical analysis and interpretation of data. MK has been involved in revising the manuscript critically for important intellectual content. MK provided final approval of the version to be published. JvH: Has made substantial contributions to the conception and design, and interpretation of the data. JvH has been involved in revising the manuscript critically for important intellectual content. JvH provided final approval of the version to be published. All authors have read and approved the final manuscript.

### Conflict of interest statement

The authors declare that the research was conducted in the absence of any commercial or financial relationships that could be construed as a potential conflict of interest.
